# Supplementary material for: Universal Pairwise Interatomic van der Waals Potentials Based on Quantum Drude Oscillators
Source: J Chem Theory Comput. 2023 Oct 24;19(21):7895–907. doi: 10.1021/acs.jctc.3c00797 (PMC10653113; doi:10.1021/acs.jctc.3c00797)
Supplement: Supplementary file 1 — ct3c00797_si_001.pdf [file ct3c00797_si_001.pdf]

Supporting Information: Universal Pairwise Interatomic van der Waals Potentials Based On Quantum Drude Oscillators

Almaz Khabibrakhmanov, Dmitry V. Fedorov, and Alexandre Tkatchenko*

Department of Physics and Materials Science, University of Luxembourg, L-1511 Luxembourg City, Luxembourg

E-mail: alexandre.tkatchenko@uni.lu

QDO damping function

Here we present the derivation of the QDO damping function given by Eq. (24) in the main manuscript, following the approach used by Tang and Toennies to derive their renowned damping function.[1,2] Asymptotic series expansion of dispersion energy is based on the assumption of non-overlapping densities. However, at typical interatomic separations in molecular systems, there is still a non-negligible overlap of electron densities. This overlap leads to short-range Pauli (exchange) repulsion, decreasing the density between interacting atoms and consequently leading to the reduced dispersion attraction between atoms, as can be easily understood using Feynman's arguments.[3] To account for this weakening of dispersion force, Tang and Toennies considered the correction to dispersion energy caused by the overlap.[1] They calculated this correction invoking the *semiclassical* Drude model,[1] whereas here we employ the *quantum* Drude oscillator model for the same purpose.

The Hamiltonian of two non-interacting identical QDOs is

$$\hat{\mathcal{H}}_0 = -\frac{\hbar^2}{2\mu}\,\boldsymbol{\nabla}^2_{\mathbf{r}_1} - \frac{\hbar^2}{2\mu}\,\boldsymbol{\nabla}^2_{\mathbf{r}_2} + \frac{k}{2}\,\mathbf{r}_1^2 + \frac{k}{2}\,\mathbf{r}_2^2 \ , \ k = \mu\omega^2 \ , \tag{S1}$$

where drudonic coordinates $\mathbf{r}_1 = (x_1, y_1, z_1)$ and $\mathbf{r}_2 = (x_2, y_2, z_2)$ have their origins, respectively, at nuclei A and B separated by distance $\mathbf{R}$. The multipolar couplings between the two non-overlapping QDOs can lead only to dispersion attraction between them. Let us now consider a coupling due to the short-range repulsion $V_{\rm rep}$, which is of given form. This repulsion is usually expressed as a function of internuclear distance R and therefore serves as the external potential for drudons arising from the symmetrization of the total wave function, as discussed in the main manuscript. To deduce the connection between interdrudonic and internuclear repulsive potentials, one can expand the former as

$$V_{\rm rep}(|\mathbf{R} + \delta\mathbf{R}|) = V_{\rm rep}(R) + \frac{dV_{\rm rep}}{dR}\Delta R + \frac{1}{2}\frac{d^2V_{\rm rep}}{dR^2}(\Delta R)^2 + \dots \ , \tag{S2}$$

where $\delta\mathbf{R} = \mathbf{r}_1 - \mathbf{r}_2$, and $\Delta R = |\mathbf{R} + \delta\mathbf{R}| - R$. Assuming that $\mathbf{R}$ is in the z direction and $R \gg |\delta\mathbf{R}|$ (the two nuclei are separated by a distance which is much larger than the range of drudons' fluctuations) and keeping terms up to the second order, we get

$$\Delta R \approx \frac{1}{2R}\left[2R(z_1 - z_2) + (y_1 - y_2)^2 + (x_1 - x_2)^2\right] \ . \tag{S3}$$

Substituting $\delta\mathbf{R}$ into Eq. (S2) and again keeping terms up to the second order, we have

$$\begin{aligned} V_{\text{rep}}(|\mathbf{R} + \delta\mathbf{R}|) \approx V_{\text{rep}}(R) + \frac{dV_{\text{rep}}(R)}{dR}(z_1 - z_2) + \\ + \frac{1}{2R}\frac{dV_{\text{rep}}(R)}{dR}\left[(x_1 - x_2)^2 + (y_1 - y_2)^2\right] + \frac{1}{2}\frac{d^2V_{\text{rep}}(R)}{dR^2}(z_1 - z_2)^2 \ . \end{aligned} \tag{S4}$$

Thus, the (approximate) Hamiltonian of two repulsion-coupled QDOs reads

$$\begin{aligned} \hat{\mathcal{H}}_{\text{int}} = -\frac{\hbar^2}{2\mu}\left(\boldsymbol{\nabla}^2_{\mathbf{r}_1} + \boldsymbol{\nabla}^2_{\mathbf{r}_2}\right) + \frac{k}{2}\left(\mathbf{r}_1^2 + \mathbf{r}_2^2\right) + V_{\text{rep}}(R) + \alpha(R)(z_1 - z_2) \ + \\ + \frac{\alpha(R)}{2R}\left[(x_1 - x_2)^2 + (y_1 - y_2)^2\right] + \frac{\beta(R)}{2}(z_1 - z_2)^2 \ , \end{aligned} \tag{S5}$$

with $\alpha(R) = dV_{\text{rep}}/dR$ and $\beta(R) = d^2V_{\text{rep}}/dR^2$ (for brevity, we omit the dependence of α and β on R, in what follows). The above Hamiltonian represents a quadratic form in drudonic coordinates, and the canonical transformation

$$\zeta_1 = \frac{z_1 - z_2}{\sqrt{2}} + \frac{\alpha\sqrt{2}}{k + 2\beta} \ , \quad \zeta_2 = \frac{z_1 + z_2}{\sqrt{2}} \ , \quad \xi_{1,2} = \frac{x_1 \mp x_2}{\sqrt{2}} \ , \quad \eta_{1,2} = \frac{y_1 \mp y_2}{\sqrt{2}} \ , \tag{S6}$$

brings it to the diagonal form

$$\hat{\mathcal{H}}_{\text{int}} = -\frac{\hbar^2}{2\mu}\left(\boldsymbol{\nabla}^2_{\tilde{\mathbf{r}}_1} + \boldsymbol{\nabla}^2_{\tilde{\mathbf{r}}_2}\right) + \left(\frac{k}{2} + \frac{\alpha}{R}\right)\left(\xi_1^2 + \eta_1^2\right) + \left(\frac{k}{2} + \beta\right)\zeta_1^2 + \frac{k}{2}\tilde{\mathbf{r}}_2^2 + V_{\text{rep}}(R) - \frac{\alpha^2}{k + 2\beta} \ , \tag{S7}$$

with $\tilde{\mathbf{r}}_1 = (\xi_1, \eta_1, \zeta_1)$, $\tilde{\mathbf{r}}_2 = (\xi_2, \eta_2, \zeta_2)$. The corresponding eigenfrequencies are

$$\omega_{\xi_1} = \omega_{\eta_1} = \sqrt{\frac{k}{\mu} + \frac{2\alpha}{\mu R}} \approx \omega \left[1 + \frac{\alpha}{kR}\right] \ , \ \omega_{\zeta_1} = \sqrt{\frac{k}{\mu} + \frac{2\beta}{\mu}} \approx \omega \left[1 + \frac{\beta}{k}\right] \ , \tag{S8}$$

and $\omega_{\xi_2} = \omega_{\eta_2} = \omega_{\zeta_2} = \omega$. We ensure that the Taylor expansion made in Eq. (S8) delivers a good approximation for a wide range of distances, as illustrated by Fig. S1. This reflects the fact that V_{rep} changes slowly compared to the harmonic potential. The interaction energy is

$$\begin{aligned} E_{\text{int}} = E_{\text{coupled}} - E_{\text{uncoupled}} = \frac{\hbar}{2}(\omega_{\xi_1} + \omega_{\eta_1} + \omega_{\zeta_1} + \omega_{\xi_2} + \omega_{\eta_2} + \omega_{\zeta_2}) \ + \ V_{\text{rep}}(R) \\ -\frac{\alpha^2}{k + 2\beta} - 6 \times \frac{\hbar\omega}{2} \ \approx \ \frac{\hbar\omega}{2}\left[\frac{2\alpha}{kR} + \frac{\beta}{k}\right] + V_{\text{rep}}(R) - \frac{\alpha^2}{k + 2\beta} \ . \end{aligned} \tag{S9}$$

Here, $V_{\text{rep}}(R)$ plays the role of external repulsive potential, whereas the remaining two terms represent the desired correction to dispersion energy caused by the presence of $V_{\text{rep}}(R)$. The second-order term $\alpha^2/(k+2\beta)$ should be omitted considering the first-order Taylor expansion for frequencies. Hence, for the (approximate) correction to dispersion energy, we finally have

$$\Delta V_{\text{disp}} = \frac{\hbar\omega}{2}\left[\frac{2\alpha}{kR} + \frac{\beta}{k}\right] \ . \tag{S10}$$

With our general expression for the QDO exchange energy $V_{\text{rep}} = Aq^2/R \exp(-\mu\omega R^2/2\hbar)$ given by Eq. (6) in the main manuscript, one can obtain

$$\alpha = \frac{dV_{\text{rep}}}{dR} = -Aq^2\left[\frac{1}{R^2} + \frac{\mu\omega}{\hbar}\right] e^{-\frac{\mu\omega}{2\hbar}R^2} \ , \tag{S11}$$

$$\beta = \frac{d^2V_{\text{rep}}}{dR^2} = Aq^2\left[\frac{2}{R^3} + \frac{\mu\omega}{\hbar}\frac{1}{R} + \left(\frac{\mu\omega}{\hbar}\right)^2 R\right] e^{-\frac{\mu\omega}{2\hbar}R^2} \ , \tag{S12}$$

$$\Delta V_{\text{disp}} = \frac{Aq^2}{2R}[(\gamma R)^2 - 1]e^{-\frac{(\gamma R)^2}{2}}, \quad \gamma = \sqrt{\frac{\mu\omega}{\hbar}} \ , \tag{S13}$$

where the atomic units are used with $k_e = 1/4\pi\varepsilon_0 = 1$, but $\hbar$ is kept explicitly in the equations. The correction ΔV is positive, since $R^2(\mu\omega/\hbar) \gg 1$ at typical internuclear distances.[4]

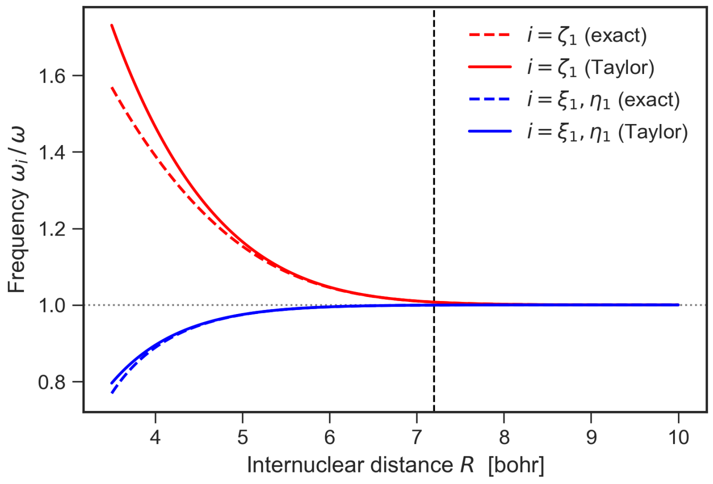

FIG. S 1: Behavior of frequencies ω_{ξ_1,η_1} (blue) and ω_{ζ_1} (red) from Eq. (S8) as a function of distance R when described by the exact square root function (dashed) and the first-order Taylor expansion (solid) at the example of Ar_2 dimer. The vertical dashed line denotes the equilibrium distance R_e.

Now we use the correction ΔV_{disp} to derive our damping function, following the approach of Tang and Toennies.[2] By introducing $B = Aq^2/2$, the sum of dispersion energy and its repulsion correction can be written as

$$-\sum_{n\geq 3} \frac{C_{2n}}{R^{2n}} + \frac{B}{R}[(\gamma R)^2 - 1]e^{-\frac{(\gamma R)^2}{2}} = -\sum_{n\geq 3} f_{2n}(R)\frac{C_{2n}}{R^{2n}} \ , \tag{S14}$$

where in the left-hand side we have incorporated the desired damping function $f_{2n}(R)$. By introducing coefficients b_{2n} such that $\sum_n b_{2n} = B$, the damping function is extracted as

$$f_{2n}(R) = 1 - \frac{b_{2n}}{C_{2n}}(\gamma^2 R^{2n+1} - R^{2n-1})e^{-\frac{(\gamma R)^2}{2}} \ . \tag{S15}$$

The coefficients b_{2n} are not uniquely defined. Therefore, from Eq. (S15) one can only assume that the general form of the damping function is

$$f_{2n}(R) = 1 - P_{2n+1}(\gamma, R)e^{-\frac{(\gamma R)^2}{2}} \ , \tag{S16}$$

where P_{2n+1} is the polynomial of the order $2n + 1$. Since damping function is dimensionless,

it can only depend on the dimensionless combination of parameters related to the considered problem, which is uniquely defined by $x = \gamma R$. Thus, we can represent damping function by

$$f_{2n}(x) = 1 - e^{-\frac{x^2}{2}} \sum_{k=0}^{2n+1} a_k x^k \ , \tag{S17}$$

To determine the polynomial coefficients a_k, we impose the natural boundary conditions

$$f_{2n}(R) \to 1 \ , \quad R \to \infty \ ; \qquad f_{2n}(R) \to 0 + O(R^{2n+1}) \ , \quad R \to 0 \ . \tag{S18}$$

Here, the first condition is fulfilled by default due to the functional form of Eq. (S17). The second condition requires that all derivatives of $f_{2n}(R)$ up to order $2n+1$ must be zero at $R = 0$, that stems from the requirement that each term $f_{2n}(R)C_{2n}/R^{2n}$ in the damped dispersion series vanishes at $R = 0$ with a finite slope.[2] By the explicit calculation of n-th derivatives, we found that, to satisfy the above boundary conditions, all odd coefficients must be zero and only even powers of x survive, resulting in the uniquely defined damping function

$$f_{2n}(z) = 1 - e^{-\frac{(\gamma R)^2}{2}} \sum_{k=0}^{n} \frac{(\gamma R)^{2k}}{2^k \cdot k!} = 1 - e^{-z} \sum_{k=0}^{n} \frac{z^k}{k!} \ , \quad z = \frac{(\gamma R)^2}{2} \ . \tag{S19}$$

This damping function has exactly the same form as the Tang-Toennies damping function[2]

$$f_{2n}(z) = 1 - e^{-z} \sum_{k=0}^{2n} \frac{z^k}{k!} \ , \quad \text{where} \ \ z = bR \ , \tag{S20}$$

with the only difference in the limits of summation, in addition to the physical meaning of unitless variable ($z = bR$ for the TT damping function, with b stemming from the Born-Mayer repulsion term Ae^{-bR}). The difference is due to the distinct form of Pauli repulsion potential between the vdW-QDO and TT models, which delivers the distinct results under applying the aforementioned boundary conditions at $R \to 0$. Interestingly, our quantum-mechanically derived damping function also matches well (but not exactly) with the one

heuristically suggested by Slipchenko and Gordon [5]

$$f_{2n}(z) = 1 - e^{-z} \sum_{k=0}^{n} \frac{z^k}{k!} \ , \quad z = (\gamma R)^2 \ , \tag{S21}$$

which they have obtained by a simple analogy to the Tang-Toennies damping function upon substitution of exponential overlap integral (of atomic densities) onto the Gaussian overlap integral (of oscillator densities). In Eq. (S21), we corrected the obvious misprint present in Ref. 5 where summation starts with $k = 1$. Another difference with our QDO damping function is that the heuristically obtained function of Slipchenko and Gordon takes $z = (\gamma R)^2$ as an argument instead of $(\gamma R)^2/2$, as obtained within our quantum-mechanical derivation.

Damped vdW-QDO potential

The derivation of the damped vdW-QDO potential is similar to the undamped one, with the only difference in the form of dispersion energy and hence dispersion force. The force-balance equation in the dipole approximation reads [4]

$$\frac{k_e q^2}{2} \left[\frac{1}{R_e^2} + \frac{\mu\omega}{\hbar} \right] e^{-\frac{\mu\omega}{2\hbar} R_e^2} = \frac{6 f_6(R_e) C_6}{R_e^7} - \frac{f_6'(R_e) C_6}{R_e^6} \ . \tag{S22}$$

Incorporating C_8 and C_{10} terms and the constant A_d , we get the full force-balance equation

$$A_d k_e q^2 \left[\frac{1}{R_e^2} + \frac{\mu\omega}{\hbar} \right] e^{-\frac{\mu\omega}{2\hbar} R_e^2} = \sum_{n=3}^{5} \left(\frac{2n f_{2n}(R_e) C_{2n}}{R_e^{2n+1}} - \frac{f_{2n}'(R_e) C_{2n}}{R_e^{2n}} \right) \ . \tag{S23}$$

Solving Eqs. (S22) and (S23) together, one can express A_d by

$$A_d = \left(\frac{1}{2} + \frac{C_8}{2 C_6 R^2} \cdot \frac{8 f_8 - R f_8'}{6 f_6 - R f_6'} + \frac{C_{10}}{2 C_6 R^4} \cdot \frac{10 f_{10} - R f_{10}'}{6 f_6 - R f_6'} \right) \bigg|_{R=R_e} \tag{S24}$$

and write the damped vdW-QDO potential as

$$V_{\text{QDO}}^{\text{damp}}(R) = \frac{A_d k_e q^2}{R} e^{-\gamma R^2/2} - \sum_{n=3}^{5} f_{2n}(R) \frac{C_{2n}}{R^{2n}} \ . \tag{S25}$$

Similar to the undamped case, we use Ne_2 to obtain the dimensionless shape of the potential

$$U_{\text{QDO}}^{\text{Ne,damp}}(x) = \frac{A_d^*}{x} e^{-\frac{(\gamma^* x)^2}{2}} - \sum_{n=3}^{5} f_{2n}(\gamma^* x) \frac{C_{2n}^*}{x^{2n}} \ , \tag{S26}$$

with the starred parameters for this equation defined in Table S 1. For other dimers, the potentials are obtained via rescaling

$$V_{\text{QDO}}^{\text{damp}}(R) = D_e U_{\text{QDO}}^{\text{Ne,damp}}(x) \ , \quad x = R/R_e \ , \tag{S27}$$

where for D_e we use the same Eq. (17) in the main text as for the undamped potential. The above derivation is not specific to noble gases remaining valid also for group II dimers. The corresponding starred parameters are listed in Table IV of the main text.

Figure S2 shows the obtained damped vdW-QDO potential curves for noble-gas dimers compared to the TTS potential[6] and reference CCSD(T) calculations. One can see almost the same curves as in the undamped case, having perfect agreement with the references. The only noticeable differences to the undamped case can be observed in the short-range region.

TABLE S 1: The dimensionless parameters in Eq. (S26). The parameters of Ne_2 dimer used in the second column are $D_e =$ 3.620 meV = 13.304×10^{-5} a.u. and $R_e = 5.875$ bohr. The value of D_e was obtained by substituting $R = R_e$ to Eq. (S25). The damped QDO parameters for Ne_2 are $q_d = 1.20299$, $\mu_d = 0.38066$, $\omega = 1.19326$ (in a.u.).

Parameter	Definition	Numerical value
A_d^*	$A_d k_e q_d^2 / R_e D_e$	1415.607
γ^*	$R_e \sqrt{\mu_d \omega / \hbar}$	3.959
C_6^*	$C_6 / D_e R_e^6$	1.1667
C_8^*	$5 C_6 / D_e R_e^6 (\gamma^*)^2$	0.3721
C_{10}^*	$245 C_6 / 8 D_e R_e^6 (\gamma^*)^4$	0.1454

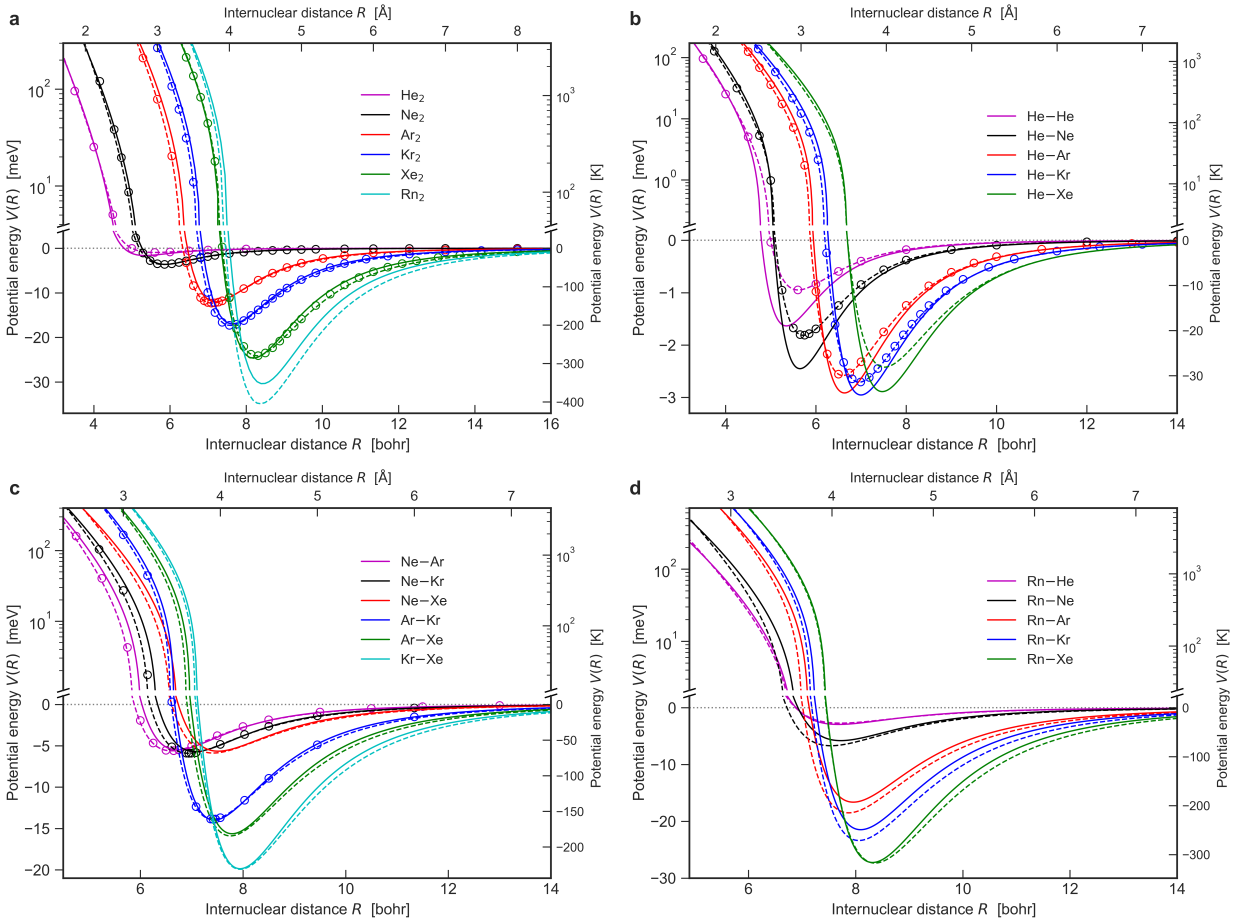

FIG. S 2: The same as Fig. 2 of the main text but for the damped vdW-QDO potential (S25).

vdW-OQDO parametrization scheme

To set the value of product $\mu\omega$, we use the condition of balance of exchange repulsion and dispersion forces between two QDOs at the equilibrium in the dipole approximation

$$\frac{k_e q^2}{2}\left[\frac{1}{R_e^2}+\frac{\mu\omega}{\hbar}\right]e^{-\frac{\mu\omega}{2\hbar}R_e^2}=\frac{6C_6}{R_e^7}\ . \tag{S28}$$

In Ref. 4, it was shown that for typical R_e in noble-gas dimers $\mu\omega/\hbar$ is an order of magnitude larger than $1/R_e^2$, and therefore the term $1/R^2$ was neglected. This well works as the first-order approximation and leads to the quantum-mechanical scaling law for vdW radius (Eq. (8) of the main manuscript). However, we found that for building the QDO-based vdW potential, this simplification introduces additional error in the location and depth of the

potential minimum by making the energies and forces inconsistent. Therefore, here we use full Eq. (S28) to rederive consistent optimized QDO parametrization in analogy with Ref. 7.

Substituting $C_6 = 3k_e^2\hbar\omega\alpha_1(q^2/\mu\omega^2)/4$ into Eq. (S28) and cancelling q^2, we obtain

$$k_e\left[\frac{\mu\omega}{\hbar}+\frac{1}{R_e^2}\right]e^{-\frac{\mu\omega}{2\hbar}R_e^2}=\frac{9\hbar}{\mu\omega}\frac{\alpha_1 k_e^2}{R_e^7}\ . \tag{S29}$$

Solving this for α_1 delivers

$$\alpha_1=(4\pi\varepsilon_0)\,e^{-\frac{\mu\omega}{2\hbar}R_e^2}\left[\left(\frac{\mu\omega}{3\hbar}\right)^2+\frac{\mu\omega}{9\hbar R_e^2}\right]R_e^7\ . \tag{S30}$$

From the other hand, for real atoms α_1 can be accurately recovered via R_e as[8]

$$\alpha_1=\frac{(4\pi\varepsilon_0)}{a_0^4}\frac{\alpha_{\rm fsc}^{4/3}}{128}R_e^7\ , \tag{S31}$$

where $\alpha_{\rm fsc}=e^2/4\pi\varepsilon_0\hbar c\approx 1/137.036$ is the fine-structure constant. Combining Eqs. (S30) and (S31), one can obtain

$$e^{-\frac{\mu\omega}{2\hbar}R_e^2}\left[\left(\frac{\mu\omega}{3\hbar}\right)^2+\frac{\mu\omega}{9\hbar R_e^2}\right]=\frac{1}{a_0^4}\frac{\alpha_{\rm fsc}^{4/3}}{128}\ . \tag{S32}$$

This equation can be rearranged and rewritten in terms of dimensionless variables

$$a\cdot e^{bx}=2x^2+\frac{x}{b}\ ,\quad x=\frac{\mu\omega a_0^2}{\hbar}\ , \tag{S33}$$

where

$$a=\frac{9\alpha_{\rm fsc}^{4/3}}{64}\ ,\quad b=\frac{R_e^2}{2a_0^2}=\frac{2(\alpha_1/4\pi\varepsilon_0)^{2/7}}{\alpha_{\rm fsc}^{8/21}a_0^{6/7}}\ . \tag{S34}$$

The above transcendental equation is similar to its counterpart from Ref. 7, and by analogy it also has two solutions, one of which is very close to zero. As discussed in Ref. 7, this almost zero solution x_B does not have a simple physical interpretation, whereas the second (larger)

root x_A of Eq. (S33) is useful to parametrize the QDO model. Thus, the full vdW-OQDO parametrization procedure can be summarized as

$$\omega = \frac{4C_6}{3\hbar\alpha_1^2 k_e^2} \ , \ \mu = \frac{\hbar x_A}{\omega a_0^2} \ , \ q = \sqrt{\alpha_1 \mu \omega^2} \ . \tag{S35}$$

The numerical values of the parameters for all noble-gas dimers are presented in Table S 3.

For the damped vdW-QDO potential, the parametrization procedure has to be adapted accordingly. The force-balance equation in dipole approximation becomes

$$\frac{k_e q^2}{2}\left[\frac{1}{R_e^2} + \frac{\mu\omega}{\hbar}\right] e^{-\frac{\mu\omega}{2\hbar}R_e^2} = \frac{6 f_6(R_e) C_6}{R_e^7} - \frac{f_6'(R_e) C_6}{R_e^6} \ . \tag{S36}$$

Repeating the manipulations performed for vdW-OQDO parametrization above, Eq. (S36) can be simplified and rewritten in terms of dimensionless variables in the form

$$a\left[e^{bx} - \left(\sum_{k=0}^{3} \frac{(bx)^k}{k!} + \frac{(bx)^4}{3\cdot 3!}\right)\right] = 2x^2 + \frac{x}{b} \ , \quad x = \frac{\mu\omega a_0^2}{\hbar} \ , \tag{S37}$$

where

$$a = \frac{18\alpha_1 a_0^4}{(4\pi\varepsilon_0)R_e^7} \ , \ b = \frac{R_e^2}{2a_0^2} \ . \tag{S38}$$

In Eq. (S 37), we used the explicit form of the QDO damping function (S 19). Eq. (S 37) has two solutions like Eq. (S 33), and we use its larger root x_A^d to set the parameters of the oscillator according to Eq. (S 35). Note that ω parameter does not change due to the damping function, since it is fully defined by α_1 and C_6.

We note that to parametrize damped vdW-QDO potential for noble-gases, Eq. (S31) can be used to make the transcendental equation (S37) dependent only on α_1. This leads to the same a and b coefficients (S34) as in vdW-OQDO case. The numerical values of the damped vdW-OQDO parameters for all noble-gas dimers are presented in Table S 3. For group II elements, however, Eq. (S 31) is not so accurate, since their dimers are not purely vdW bonded. Therefore, for group II dimers both α_1 and R_e should be used explicitly in Eq. (S

38). To construct the vdW-QDO potential for these systems, we used only the oscillator parameters for Sr_2. Nevertheless, for completeness, the damped vdW-OQDO parameters for all group II dimers are presented in Table S 2.

TABLE S 2: The damped quantum Drude oscillator parameters (in a.u.) obtained for group II dimers with the reference $\{\alpha_1, C_6\}$ from Table III of the main text.

Dimer	ω	μ_d	q_d
Mg_2	0.16445	1.10568	1.46011
Ca_2	0.11458	1.17281	1.55535
Sr_2	0.10639	1.06709	1.54334
Ba_2	0.09198	1.06133	1.56703
Zn_2	0.32010	0.65638	1.61268
Cd_2	0.43226	0.46174	1.99216
Hg_2	0.45481	0.50101	1.87435

TABLE S 3: Effective polarizabilities (α_1^{AB}) and dispersion coefficients (C_6^{AB}) of noble-gas dimers obtained according to Eqs. (19) and (21) of the main text. The corresponding parameters of the quantum Drude oscillator obtained within the vdW-OQDO parametrization procedure are also shown. Parameter sets $\{\omega, \mu, q\}$ and $\{\omega, \mu_d, q_d\}$ correspond to the vdW-QDO potential without and with damping function, respectively. All values are in a.u.

Dimer	α_1^{AB}	C_6^{AB}	ω	μ	q	μ_d	q_d
He–He	1.38	1.46	1.02219	0.55810	0.89707	0.56760	0.90468
He–Ne	2.025	3.043	0.98941	0.49841	0.99400	0.50884	1.00434
He–Ar	6.24	9.513	0.32574	0.97613	0.80393	1.01360	0.81921
He–Kr	9.09	13.316	0.21487	1.27205	0.73066	1.33196	0.74767
He–Xe	14.34	19.265	0.12491	1.81468	0.63720	1.92445	0.65619
He–Rn	17.46	23.265	0.10175	2.05164	0.60901	2.19004	0.62922
Ne–Ne	2.67	6.38	1.19326	0.37164	1.18865	0.38066	1.20299
Ne–Ar	6.885	19.539	0.54960	0.55625	1.07555	0.57877	1.09710
Ne–Kr	9.735	27.225	0.38304	0.69398	0.99559	0.72791	1.01964
Ne–Xe	14.985	39.144	0.23243	0.95753	0.88044	1.01687	0.90731
Ne–Rn	18.105	47.232	0.19212	1.07013	0.84567	1.14379	0.87429
Ar–Ar	11.1	64.3	0.69583	0.36208	1.39498	0.38110	1.43115
Ar–Kr	13.95	91.100	0.62418	0.36734	1.41296	0.38922	1.45444
Ar–Xe	19.2	133.995	0.48465	0.41383	1.36611	0.44326	1.41386
Ar–Rn	22.32	162.193	0.43409	0.43337	1.35007	0.46689	1.40132
Kr–Kr	16.8	129.6	0.61224	0.34654	1.47727	0.36943	1.52526
Kr–Xe	22.05	191.715	0.52575	0.35968	1.48061	0.38732	1.53644
Kr–Rn	25.17	232.246	0.48879	0.36552	1.48258	0.39578	1.54272
Xe–Xe	27.3	285.9	0.51148	0.33725	1.55198	0.36648	1.61783
Xe–Rn	30.42	346.742	0.49960	0.32938	1.58144	0.35973	1.65270
Rn–Rn	33.54	420.6	0.49852	0.31624	1.62358	0.34706	1.70084

Errors for two different parametrizations of Lennard-Jones potential

In this section, we report the Lennard-Jones parameters and errors of the corresponding LJ potentials for noble-gas dimers. We considered two sets of LJ parameters. The first one, denoted as LJ1 throughout the manuscript, is taken from Ref. 9, where $(\sigma_1, \varepsilon_1)$ are compiled from different sources. These parameters were fitted to reproduce the thermodynamical data for noble gases. The second set of parameters, denoted as LJ2, was created by ourselves. We used the reference *ab initio* values of R_e and D_e compiled in Ref. 6 to determine $(\sigma_2, \varepsilon_2)$ using the obvious relations $\sigma_2 = R_e \times 2^{-1/6}$, $\varepsilon_2 = D_e$. The numerical values for both parameter sets are presented in Table S 4.

TABLE S 4: Two considered sets of Lennard-Jones parameters for noble gases: LJ1 $(\sigma_1, \varepsilon_1)$[9] and LJ2 $(\sigma_2, \varepsilon_2)$ obtained by ourselves. The units are bohr for σ and meV for ε.

Element	σ_1	ε_1	σ_2	ε_2
He	4.838	0.8807	4.996	0.9475
Ne	5.197	3.0678	5.194	3.6324
Ar	6.425	10.341	6.334	12.319
Kr	6.803	14.736	6.761	17.310
Xe	7.748	18.958	7.370	24.126

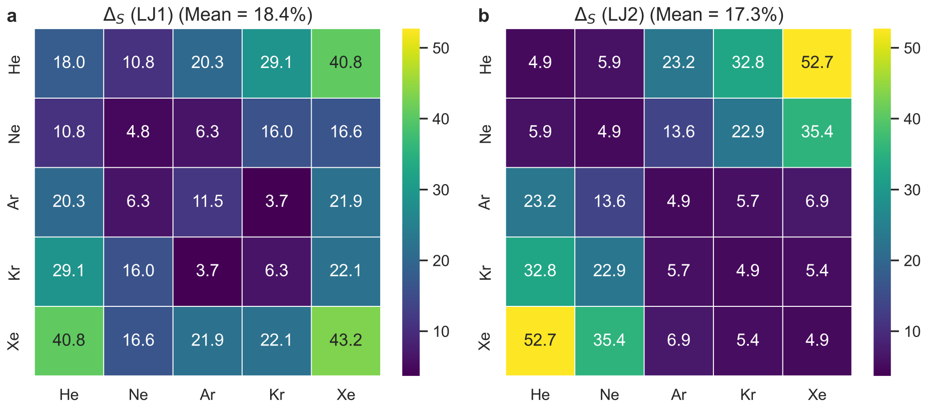

FIG. S 3: Δ_S metric (in %) calculated for two LJ parametrizations with respect to the TTS potential.[6]

In Figure S3 we show the error of LJ1 and LJ2 potentials expressed in terms of the Δ_S metrics, as described in the main text. As one can see, the LJ2 parametrization, designed to reproduce correct $\{R_e, D_e\}$, works well for dimers composed of atoms possessing similar vdW radius (He, Ne is one group, and Ar, Kr, Xe is another). However, for dimers mixed between

the two groups, errors of LJ2 increase drastically. This indicates the lack of flexibility of the LJ potential as well as limited accuracy of Lorentz-Berthelot combination rules.

Relation between the vdW-QDO and TT models and SAPT

Within the SAPT framework,[10,11] the interaction energy of two noble-gas atoms can be represented as the sum of four contributions

$$E_{\text{int}}^{\text{SAPT}} = E_{\text{elst}}^{(1)} + E_{\text{exc}}^{(1)} + E_{\text{disp}}^{(2)} + E_{\text{exc-disp}}^{(2)} \,, \tag{S39}$$

since induction and exchange-induction terms perfectly cancel out each other delivering $E_{\text{ind}}^{(2)} + E_{\text{exc-ind}}^{(2)} \approx 0$ (see Figure S4d). On the other hand, Tang-Toennies model relies on a more compact decomposition of the interaction energy, consisting only of exchange repulsion and dispersion. Nevertheless, the total interaction energies obtained by means of the TT model and SAPT are practically the same for noble-gas dimers. This interesting fact has been already discussed in literature.[12] Similar to the TT model, our vdW-QDO potential also relies on the balance between exchange repulsion and dispersion. Therefore, we find it useful to report here a simple analysis, which elucidates the reasons behind the high accuracy of the vdW-QDO and TT models.

In Figure S4a-b, we separately compare the dispersion and exchange repulsion terms of vdW-QDO and TT potentials for neon dimer. A close agreement between the undamped vdW-QDO (red solid curve) and TT (blue curve) potentials is observed for both terms, with the discrepancy appearing more pronounced only at shorter distances. In contrast, the damped vdW-QDO dispersion and exchange repulsion (red dashed curves) are significantly different, although their sum shows a perfect agreement with undamped vdW-QDO and TT potentials (Figure S4c). In addition, we compare the energy terms of vdW-QDO and TT models to the corresponding energy contributions from the SAPT-CCSD calculations[14] (black solid curves). A noticeable difference for both dispersion and exchange repulsion energies can be spotted. In other words, the dispersion energy from the vdW-QDO and TT

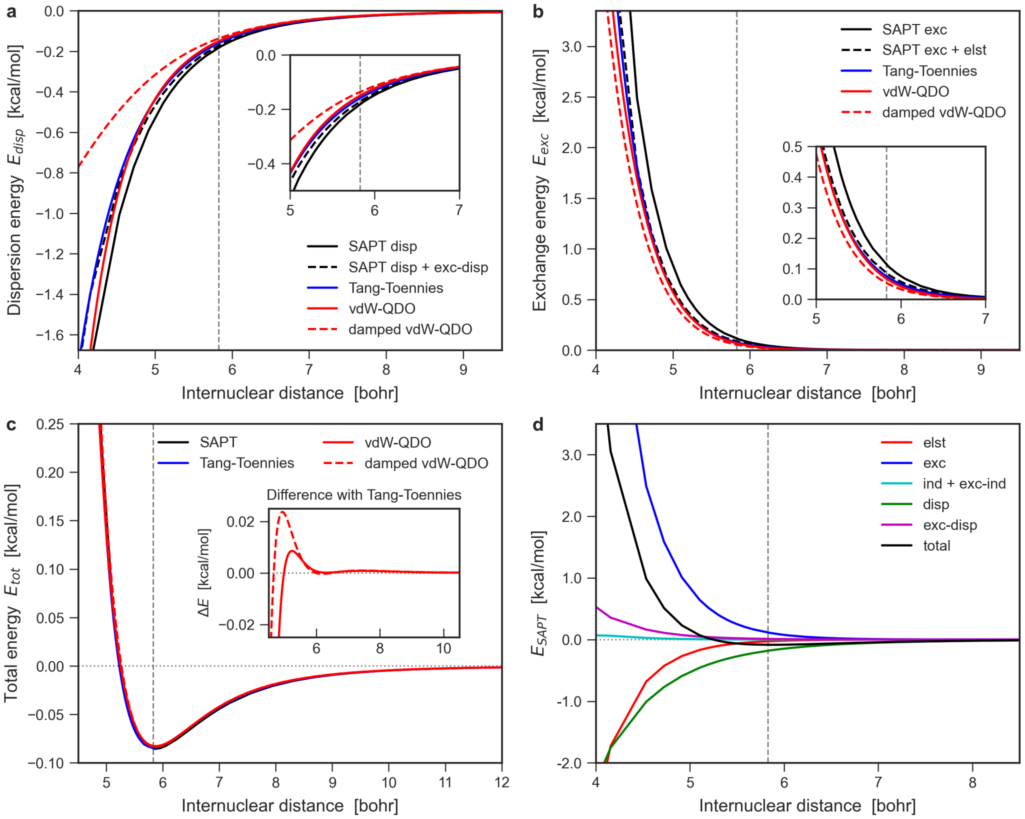

FIG. S4: (a) Dispersion and (b) exchange repulsion components of the vdW-QDO (red) and Tang-Toennies [13] (blue) potentials compared to the SAPT energy contributions (black) for neon dimer. The comparison of total interaction curves is shown in (c). The SAPT data was taken from Ref. 14. Individual SAPT contributions for neon are displayed in (d). The insets in (a) and (b) show zoom-in to near-equilibrium distances. The inset in (c) is the discrepancy between Tang-Toennies potential and undamped/damped vdW-QDO potentials. Everywhere the vertical dashed line denotes the equilibrium distance.

potentials does not *exactly* reproduce $E^{(2)}_{\rm disp}$ curve from SAPT, and the same is true also for their exchange repulsion parts versus $E^{(1)}_{\rm exc}$. However, if instead one compares to the sums $E^{(1)}_{\rm elst} + E^{(1)}_{\rm exc}$ and $E^{(2)}_{\rm disp} + E^{(2)}_{\rm exc-disp}$, then much closer agreement with the vdW-QDO and TT energy decomposition is observed, as illustrated by black dashed lines.

Thus, we conclude that both vdW-QDO and TT potentials rely on the effective description of exchange repulsion and electrostatics by the *exchange term* and on the effective description of dispersion and exchange-dispersion interactions by the *dispersion term* in the potential. We believe that this observation is important, since it allows to embed the designed accurate interatomic potentials into the physical context of non-covalent interactions picture, which is rarely done.

vdW-QDO potentials for the molecular dimers

Here we present our results for the eight molecular dimers considered in our study. They allow us to conclude that the vdW-QDO potential works well in predicting dispersion energy between aliphatic molecules, but it has difficulties with treating benzene. We attribute this to a high anisotropy of benzene polarizability tensor (longitudinal component is twice as large as the transverse one[15]), which means that dispersion interaction requires many-body treatment in this case.

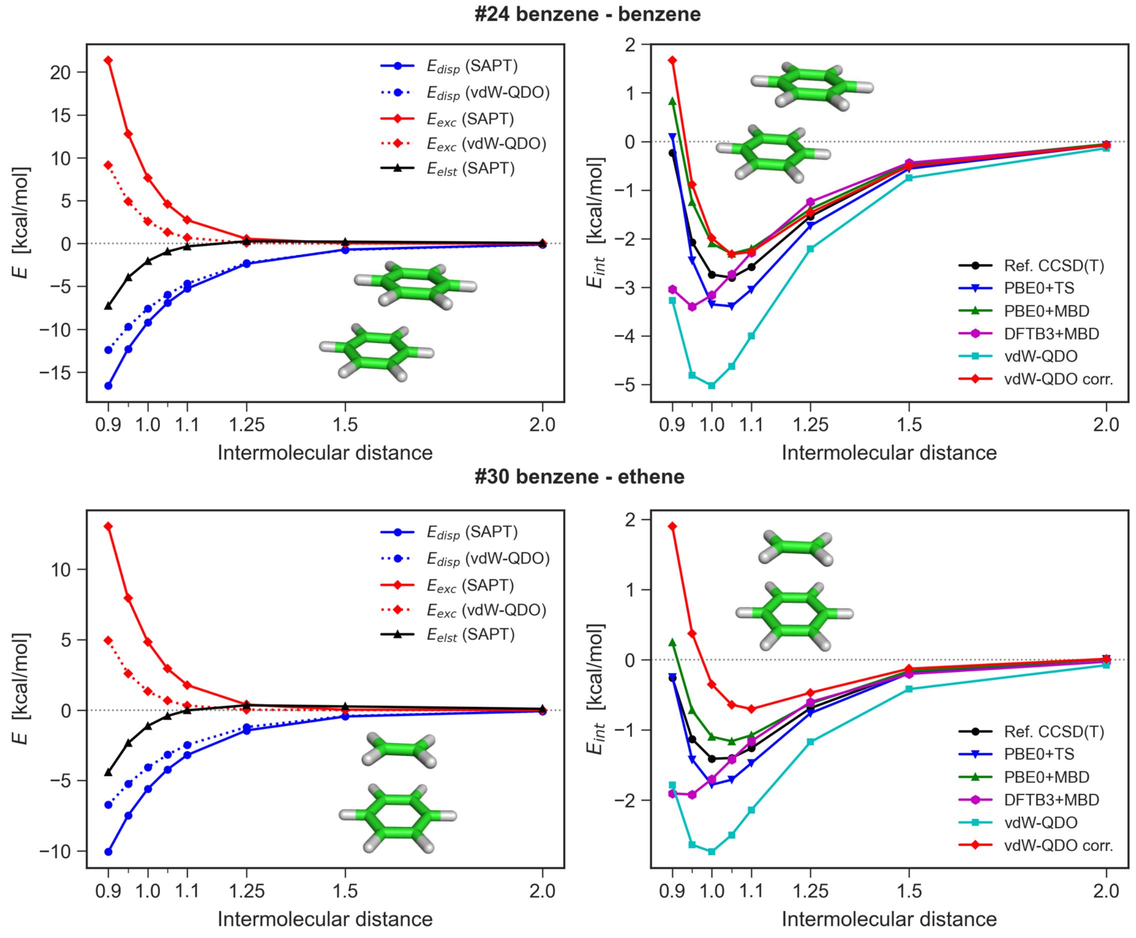

FIG. S 5: **Left:** Dispersion (blue) and exchange (red) contributions to the interaction energy of molecular dimers (shown as insets) calculated by SAPT-DFT(solid lines) and damped vdW-QDO potential (dotted lines). In addition, electrostatic term from SAPT-DFT is displayed in black. The presented SAPT-DFT results correspond to the calculations from Ref. 16 with the ALDA xc-kernel and pVQZ basis sets. **Right:** Interaction energy curves of molecular dimers as calculated by different methods: reference CCSD(T)[17] (black circles); PBE0+TS (blue) and PBE0+MBD (green) corrections; DFTB3+MBD (magenta); damped vdW-QDO potential (cyan); SAPT-corrected vdW-QDO potential (red).

We also observe that the accuracy of the vdW-QDO method is higher for more spherical molecules. The best results for dispersion energy are obtained for the dimer of neopentane, which is the closest to the spherical symmetry among the considered molecules.

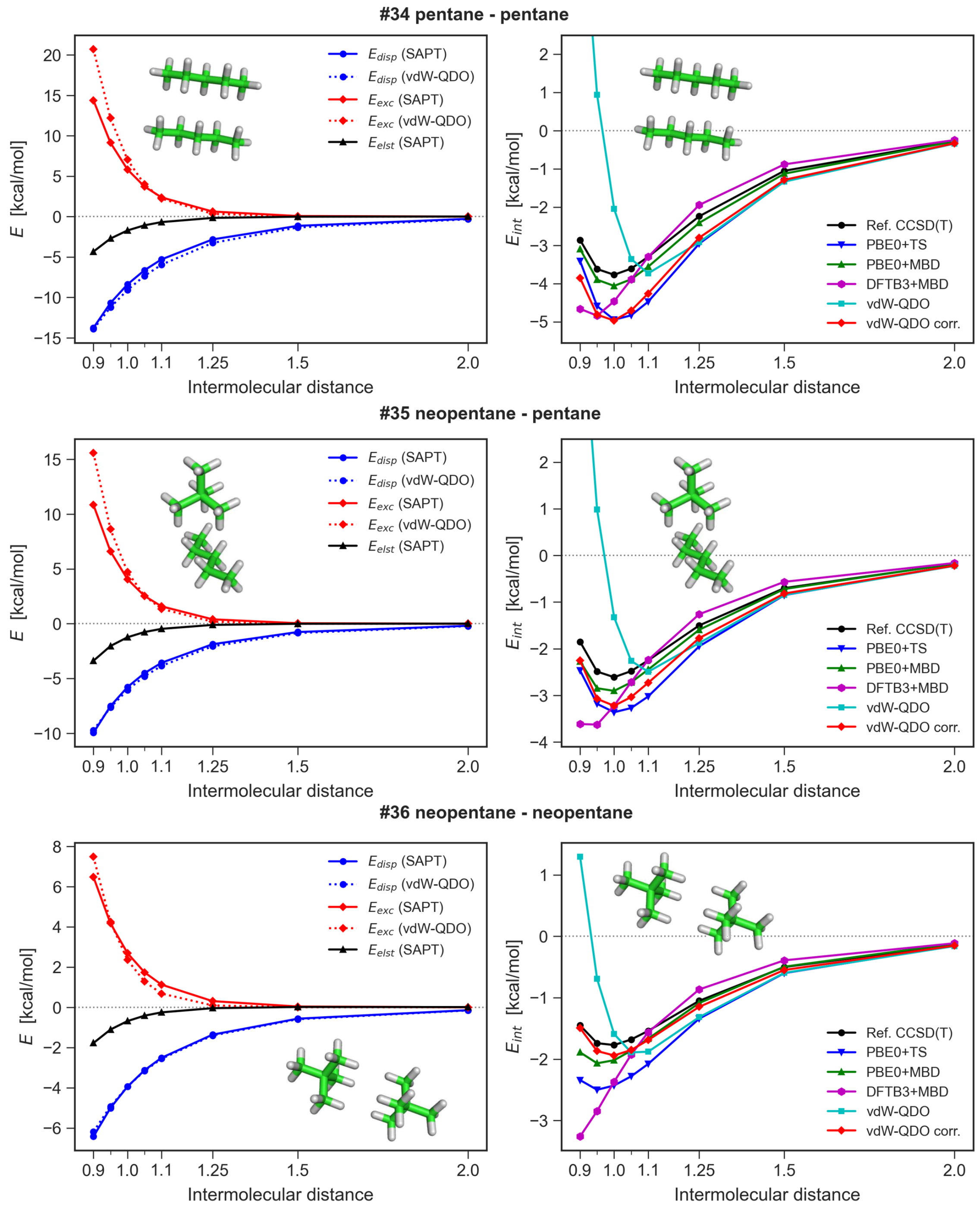

FIG. S 6: The same as Figure S5 for pentane, neopentane-pentane, and neopentane dimers.

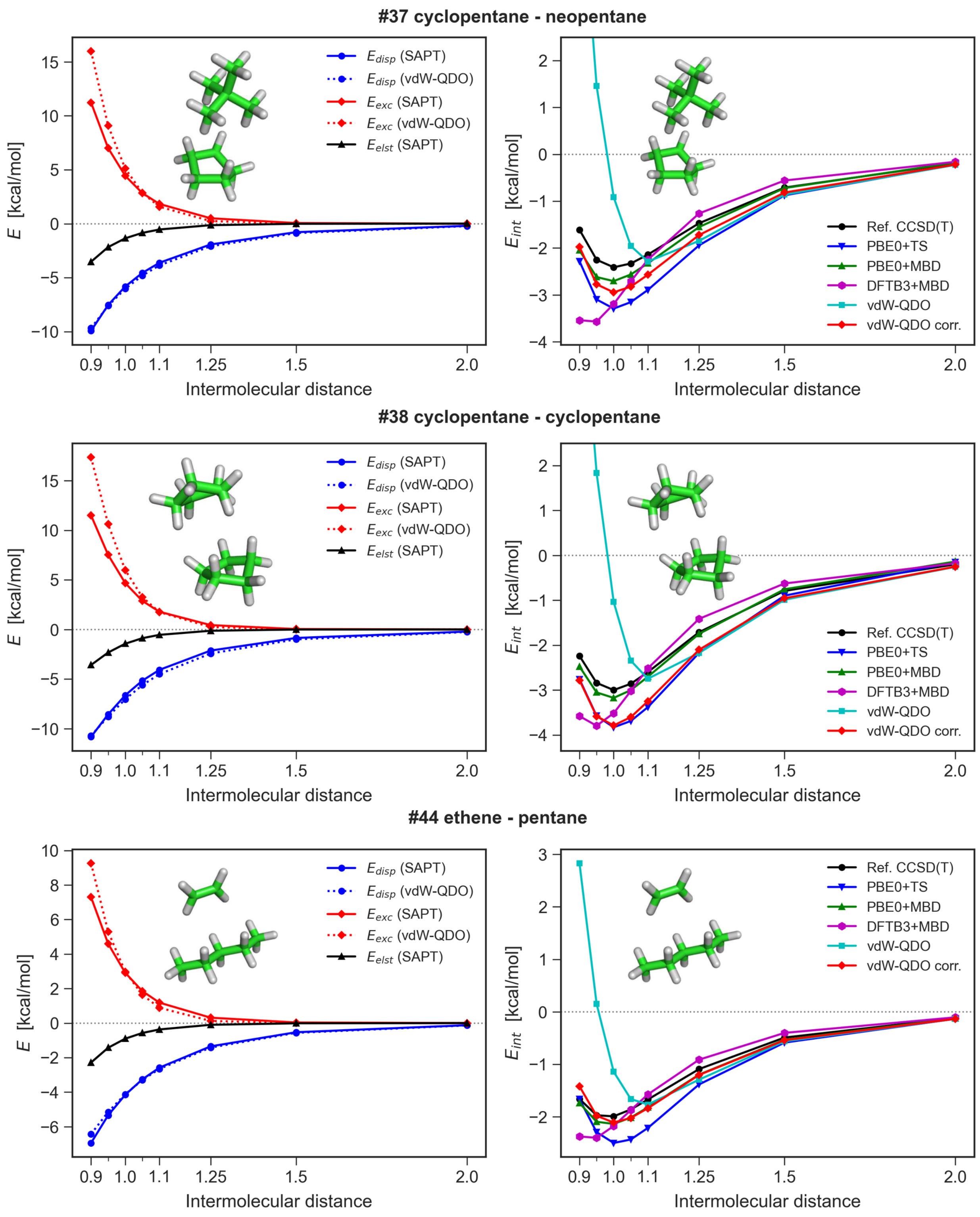

FIG. S7: The same as Figure S5 for cyclopentane-neopentane, cyclopentane, and ethene-pentane dimers.

References

(1) Tang, K. T.; Toennies, J. P. A simple theoretical model for the van der Waals potential at intermediate distances. I. Spherically symmetric potentials. *J. Chem. Phys.* **1977**, *66*, 1496–1506.

(2) Tang, K. T.; Toennies, J. P. An improved simple model for the van der Waals potential based on universal damping functions for the dispersion coefficients. *J. Chem. Phys.* **1984**, *80*, 3726–3741.

(3) Feynman, R. P. Forces in molecules. *Phys. Rev.* **1939**, *56*, 340–343.

(4) Fedorov, D. V.; Sadhukhan, M.; Stöhr, M.; Tkatchenko, A. Quantum-Mechanical Relation between Atomic Dipole Polarizability and the van der Waals Radius. *Phys. Rev. Lett.* **2018**, *121*, 183401.

(5) Slipchenko, L. V.; Gordon, M. S. Damping functions in the effective fragment potential method. *Mol. Phys.* **2009**, *107*, 999–1016.

(6) Sheng, X.; Toennies, J. P.; Tang, K. Conformal Analytical Potential for All the Rare Gas Dimers over the Full Range of Internuclear Distances. *Phys. Rev. Lett.* **2020**, *125*, 253402.

(7) Góger, S.; Khabibrakhmanov, A.; Vaccarelli, O.; Fedorov, D. V.; Tkatchenko, A. Optimized Quantum Drude Oscillators for Atomic and Molecular Response Properties. *J. Phys. Chem. Lett.* **2023**, *14*, 6217–6223.

(8) Tkatchenko, A.; Fedorov, D. V.; Gori, M. Fine-Structure Constant Connects Electronic Polarizability and Geometric van-der-Waals Radius of Atoms. *J. Phys. Chem. Lett.* **2021**, *12*, 9488–9492.

(9) Mamedov, B. A.; Somuncu, E. Analytical treatment of second virial coefficient over

Lennard-Jones (2n - n) potential and its application to molecular systems. *J. Mol. Struct.* **2014**, *1068*, 164–169.

(10) Jeziorski, B.; Moszynski, R.; Szalewicz, K. Perturbation theory approach to intermolecular potential energy surfaces of van der Waals complexes. *Chem. Rev.* **1994**, *94*, 1887–1930.

(11) Szalewicz, K.; Jeziorski, B. Physical mechanisms of intermolecular interactions from symmetry-adapted perturbation theory. *J. Mol. Model.* **2022**, *28*, 273.

(12) Tang, K. T.; Toennies, J. P.; Yiu, C. L. The generalized Heitler-London theory for interatomic interaction and surface integral method for exchange energy. *Int. Rev. Phys. Chem.* **1998**, *17*, 363–406.

(13) Tang, K. T.; Toennies, J. P. The van der Waals potentials between all the rare gas atoms from He to Rn. *J. Chem. Phys.* **2003**, *118*, 4976–4983.

(14) Shirkov, L.; Sladek, V. Benchmark CCSD-SAPT study of rare gas dimers with comparison to MP-SAPT and DFT-SAPT. *J. Chem. Phys.* **2017**, *147*, 174103.

(15) Hermann, J.; DiStasio Jr., R. A.; Tkatchenko, A. First-Principles Models for van der Waals Interactions in Molecules and Materials: Concepts, Theory, and Applications. *Chem. Rev.* **2017**, *117*, 4714–4758.

(16) Heßelmann, A. DFT-SAPT intermolecular interaction energies employing exact-exchange Kohn–Sham response methods. *J. Chem. Theory Comput.* **2018**, *14*, 1943–1959.

(17) Řezáč, J.; Riley, K. E.; Hobza, P. S66: A well-balanced database of benchmark interaction energies relevant to biomolecular structures. *J. Chem. Theory Comput.* **2011**, *7*, 2427–2438.
